# Supplementary material for: L-Type Amino Acid Transporter 1 Regulates Cancer Stemness and the Expression of Programmed Cell Death 1 Ligand 1 in Lung Cancer Cells
Source: Int J Mol Sci. 2021 Oct 11;22(20):10955. doi: 10.3390/ijms222010955 (PMC8537563; doi:10.3390/ijms222010955)
Supplement: Supplementary file 1 [file ijms-22-10955-s001.zip › ijms-1372412-supplementary.pdf]

Table S1. Primers sequences used in this study.

Gene name	Primer sequence (5' to 3')
BMI1-F	5'-AATCCCCACCTGATGTGTGT-3'
BMI1-R	5'-GCTGGTCTCCAGGTAACGAA-3'
SOX2-F	5'-GCTACAGCATGATGCAGGACCA-3'
SOX2-R	5'-TCTGCGAGCTGGTCATGGAGTT-3'
OCT4-F	5'-GGTCCGAGTGTGGTTCTGTA-3'
OCT4-R	5'-CGAGGAGTACAGTGCAGTGA-3'
c-MYC-F	5'-AATGAAAAGGCCCCCAAGGTAGTTATCC-3'
c-MYC-R	5'-GTCGTTTCCGCAACAAGTCCTCTTC-3'
SLC7A5-F	5'-ACCCACCACAACAAGCAAGT-3'
SLC7A5-R	5'-CATTACTCGGCAGAGGGTGT-3'
PDL1-F	5'-TATGGTGGTGCCGACTACAA-3'
PDL1-R	5'-CTCTTGGAATTGGTGGTGGT-3'
EZH2-F	5'-GACTGGCGAAGAGCTGTTTT-3'
EZH2-R	5'-TCTTTCGATGCCGACATACTT-3'
JAG1-F	5'-GACTCATCAGCCGTGTCTCA-3'
JAG1-R	5'-TGGGGAACACTCACACTCAA-3'
IGFBP3-F	5'-GTGGATCCCTCAACCAAGAA-3'
IGFBP3-R	5'-TAGGTTCCCAGAGTGCCCTA-3'
CAV1-F	5'-CCAAGGAGATCGACCTGGTCAA-3'
CAV1-R	5'-GCCGTCAAAACTGTGTGTCCCT-3'
TGFB2-F	5'-AAGAAGCGTGCTTTGGATGCGG-3'
TGFB2-R	5'-ATGCTCCAGCACAGAAGTTGGC-3'
WEE1-F	5'-GATGTGCGACAGACTCCTCAAG-3'
WEE1-R	5'-CTGGCTTCCATGTCTTCACCAC-3'
TYMS-F	5'-GGTGTTTTGGAGGAGTTGCTGTG-3'
TYMS-R	5'-GGAGAATCCCAGGCTGTCCAAA-3'
KLF4-F	5'-CATCTCAAGGCACACCTGCGAA-3'
KLF4-R	5'-TCGGTCGCATTTTTGGCACTGG-3'
CDC25A-F	5'-TCTGGACAGCTCCTCTCGTCAT-3'
CDC25A-R	5'-ACTTCCAGGTGGAGACTCCTCT-3'
PDCD4-F	5'-ACTGTGCCAACCAGTCCAAAGG-3'
PDCD4-R	5'-CCTCCACATCATACACCTGTCC-3'
GPX3-F	5'-TACGGAGCCCTCACCATTGATG-3'
GPX3-R	5'-CAGACCGAATGGTGCAAGCTCT-3'
mRPL19-F	5'-GGGATTTGCATTCAGAGATCAG-3'
mRPL19-R	5'-GGAAGGGCATCTCGTAAG-3'
β-actin-F	5'-CAGCTCACCATGGATGATGAT-3'

β-actin-R	5’-CTCGGCCGTGGTGGTGAAGCT-3’

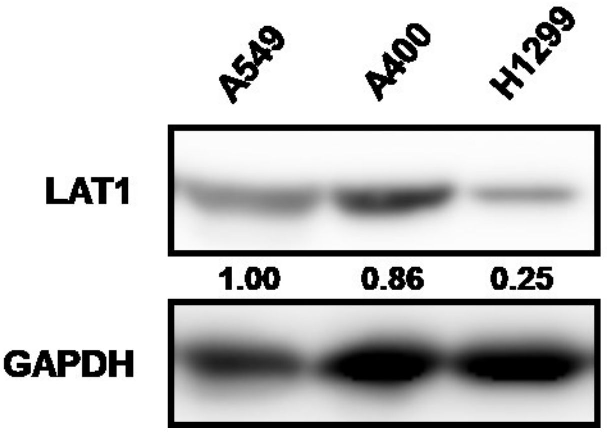

Figure S1. The total expression level of LAT1 protein among NSCLC cells used in this study.

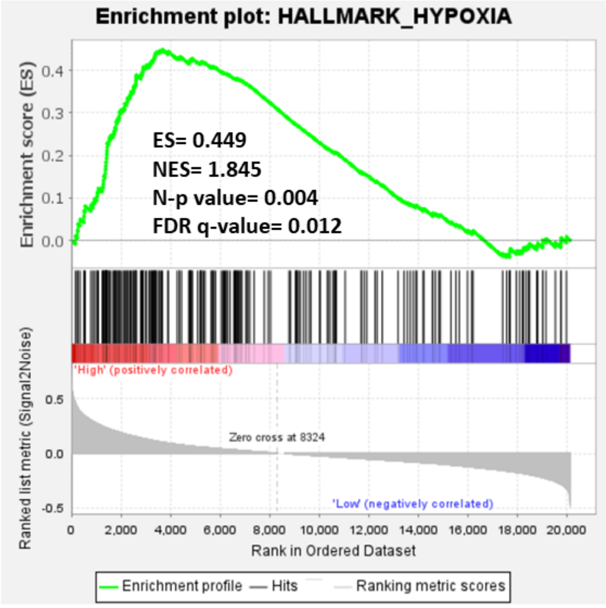

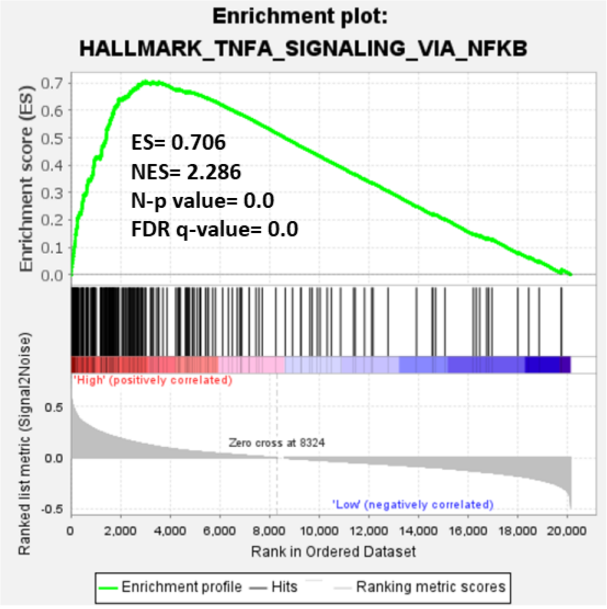

Figure S2. Gene Set Enrichment Analysis of NSCLC patients in the TCGA database according to PD-L1 expression level.

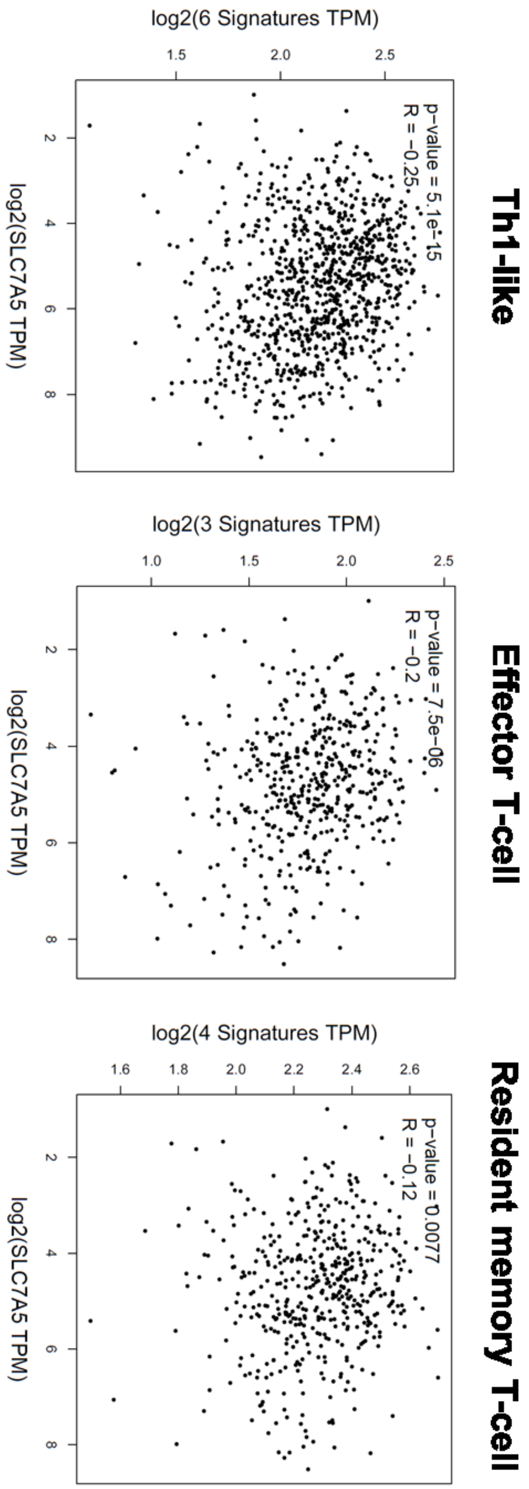

Figure S3. SLC7A5 is negatively correlated with T-cell signatures in NSCLC patients of TCGA database.

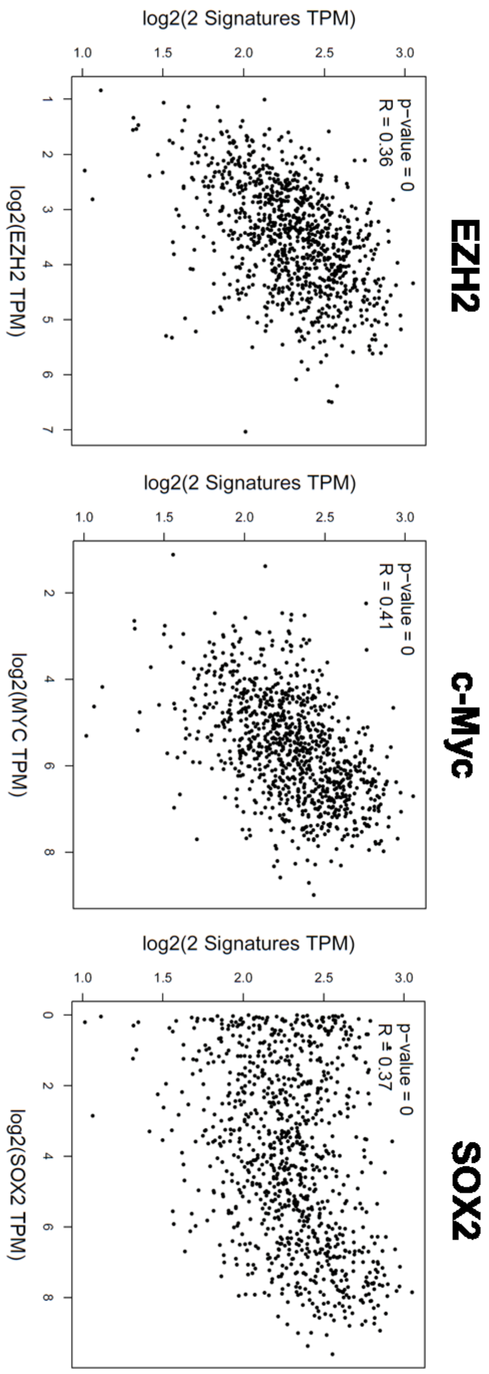

Figure S4. EZH2, c-Myc, or SOX2 is positively correlated with SLC7A5/CD274 signature in NSCLC patients of TCGA database.

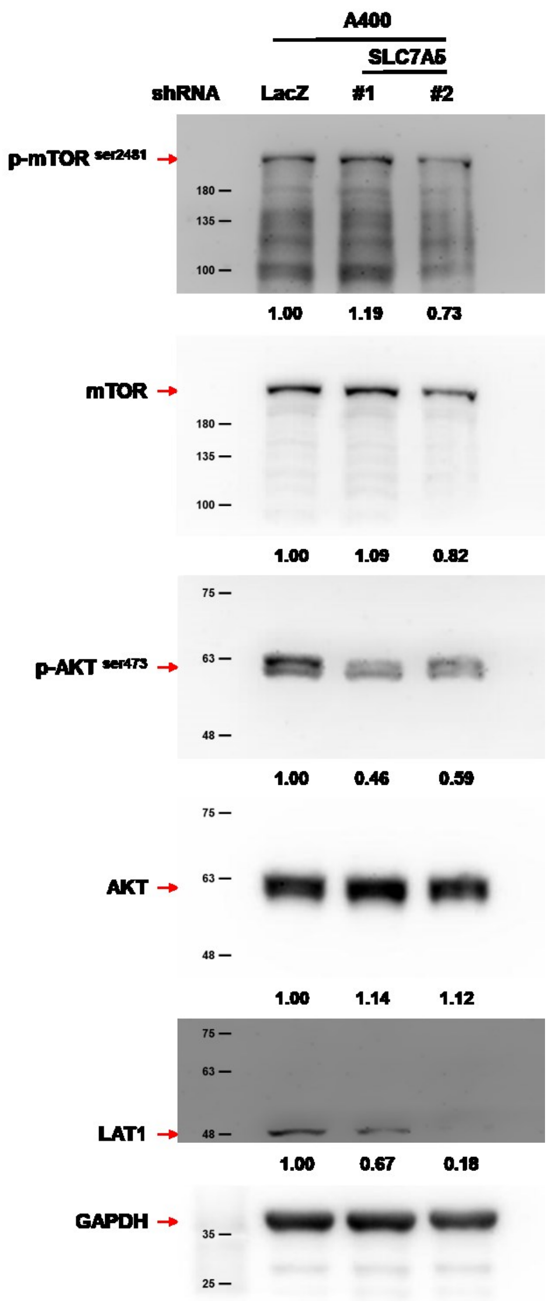

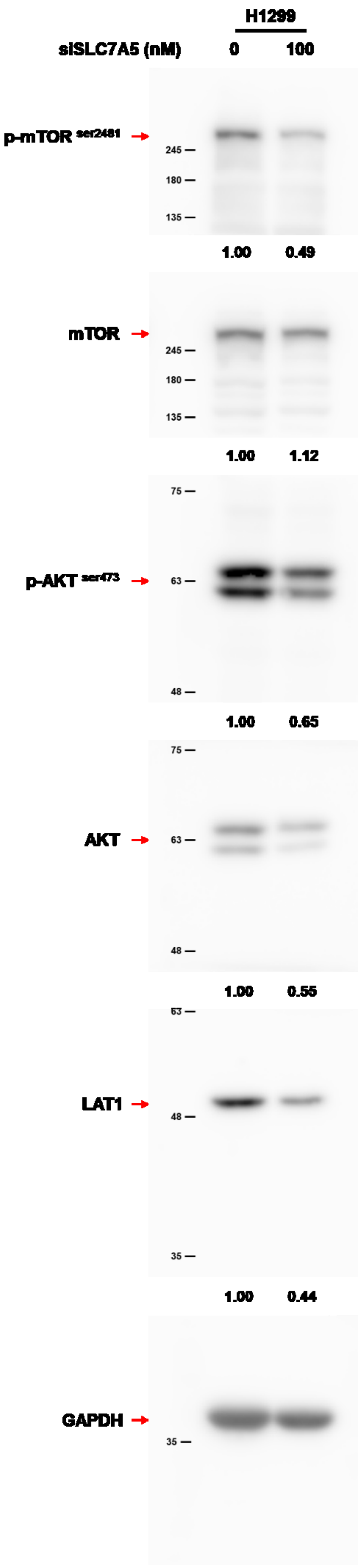

Figure S5. The original blot images of Figure 2C.

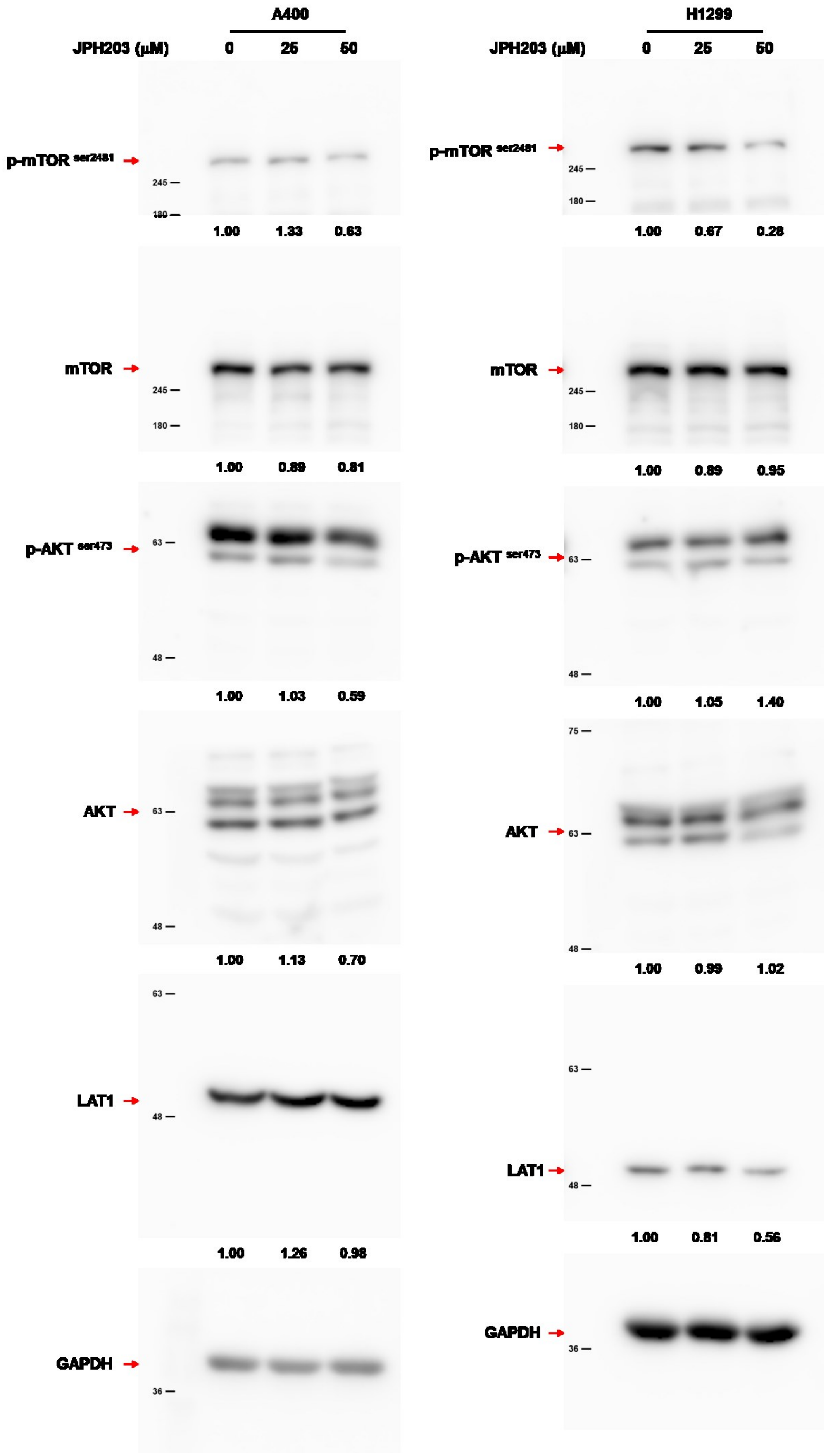

Figure S6. The original blot images of Figure 3B.

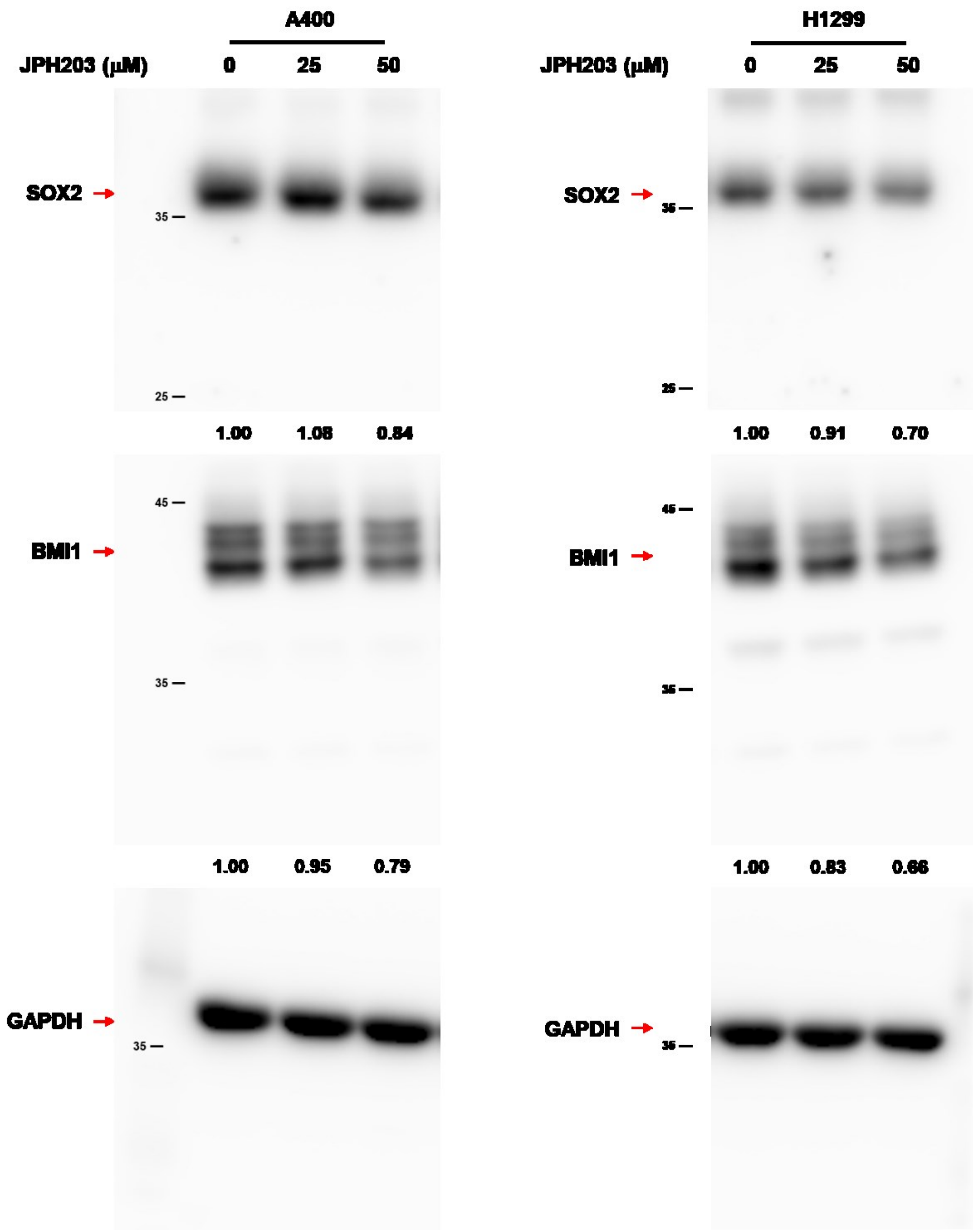

Figure S7. The original blot images of Figure 3C.

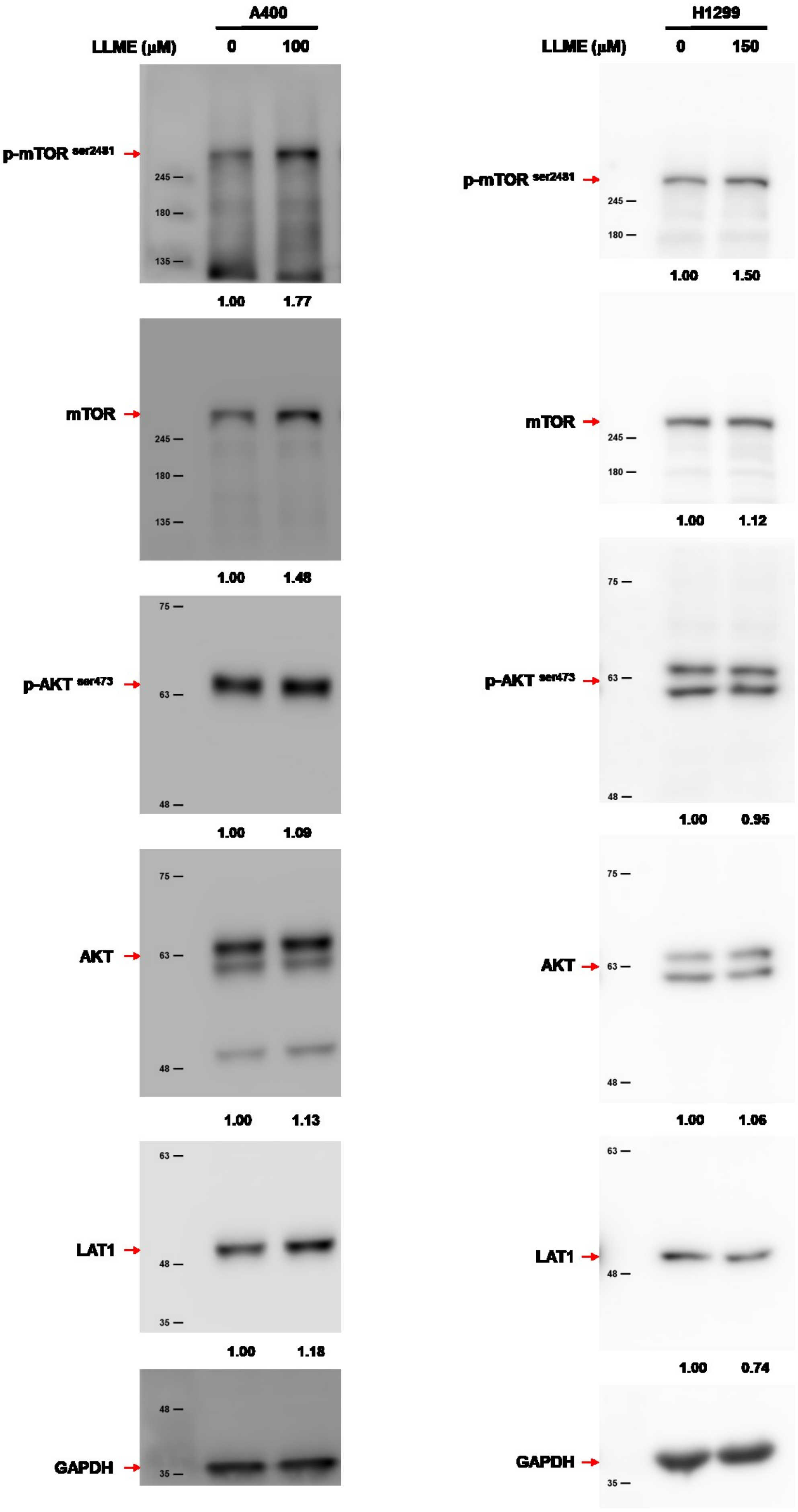

Figure S8. The original blot images of Figure 3E.

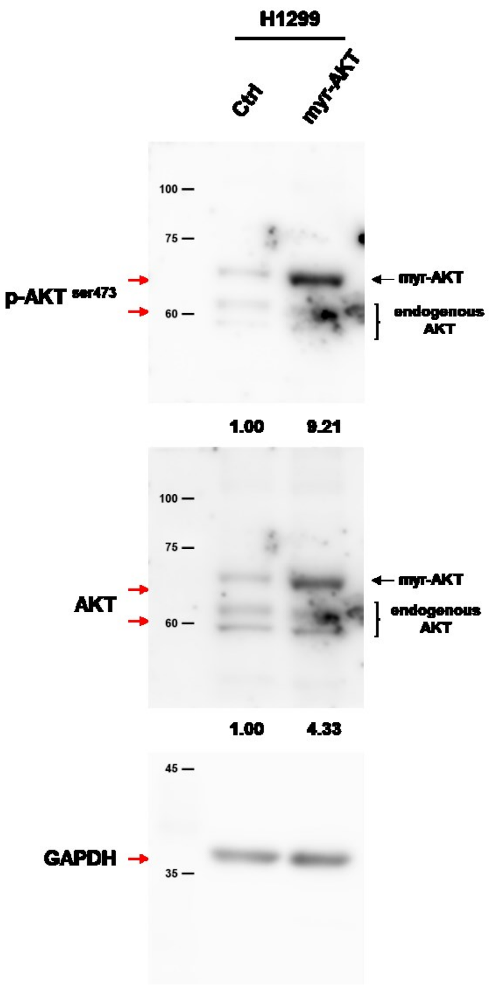

Figure S9. The original blot images of Figure 3F.

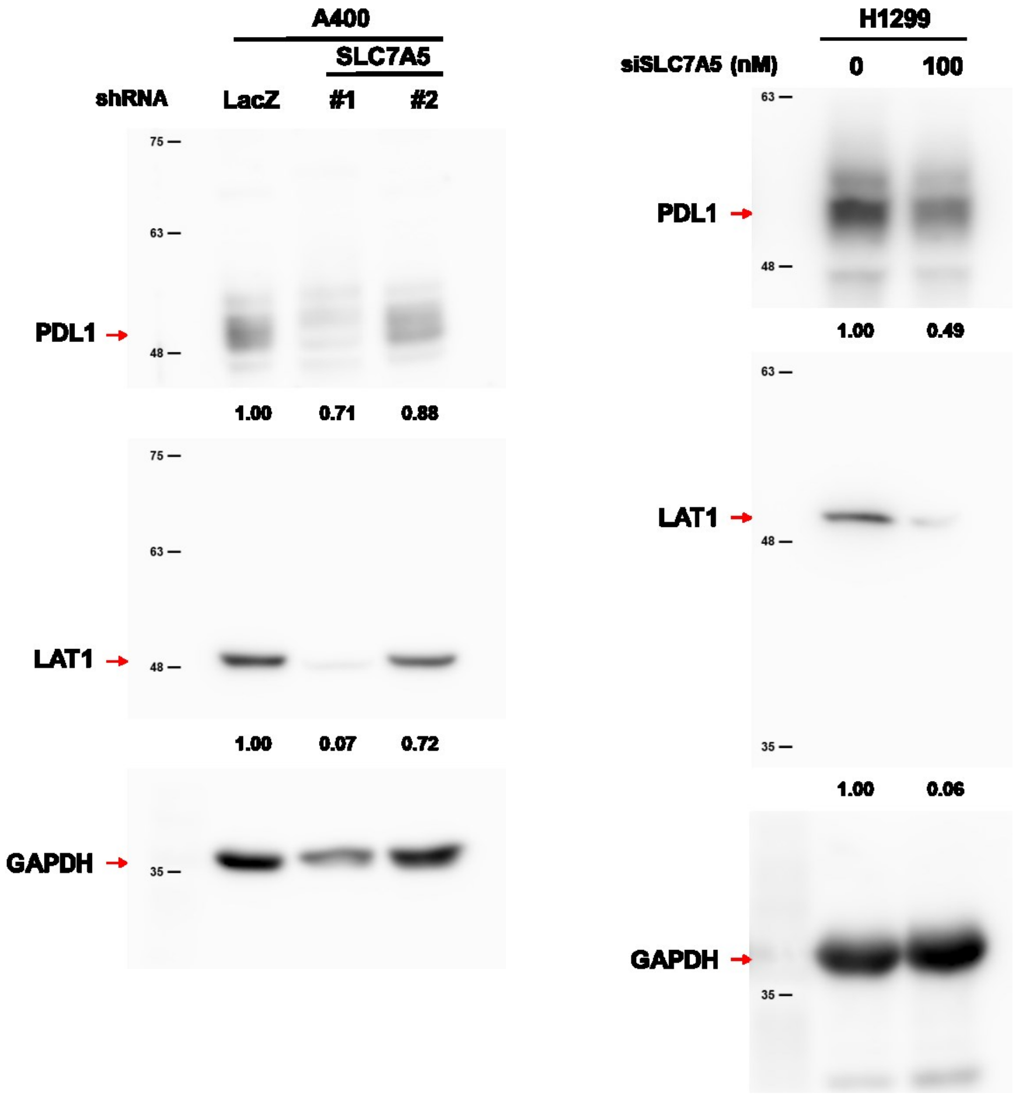

Figure S10. The original blot images of Figure 4A.

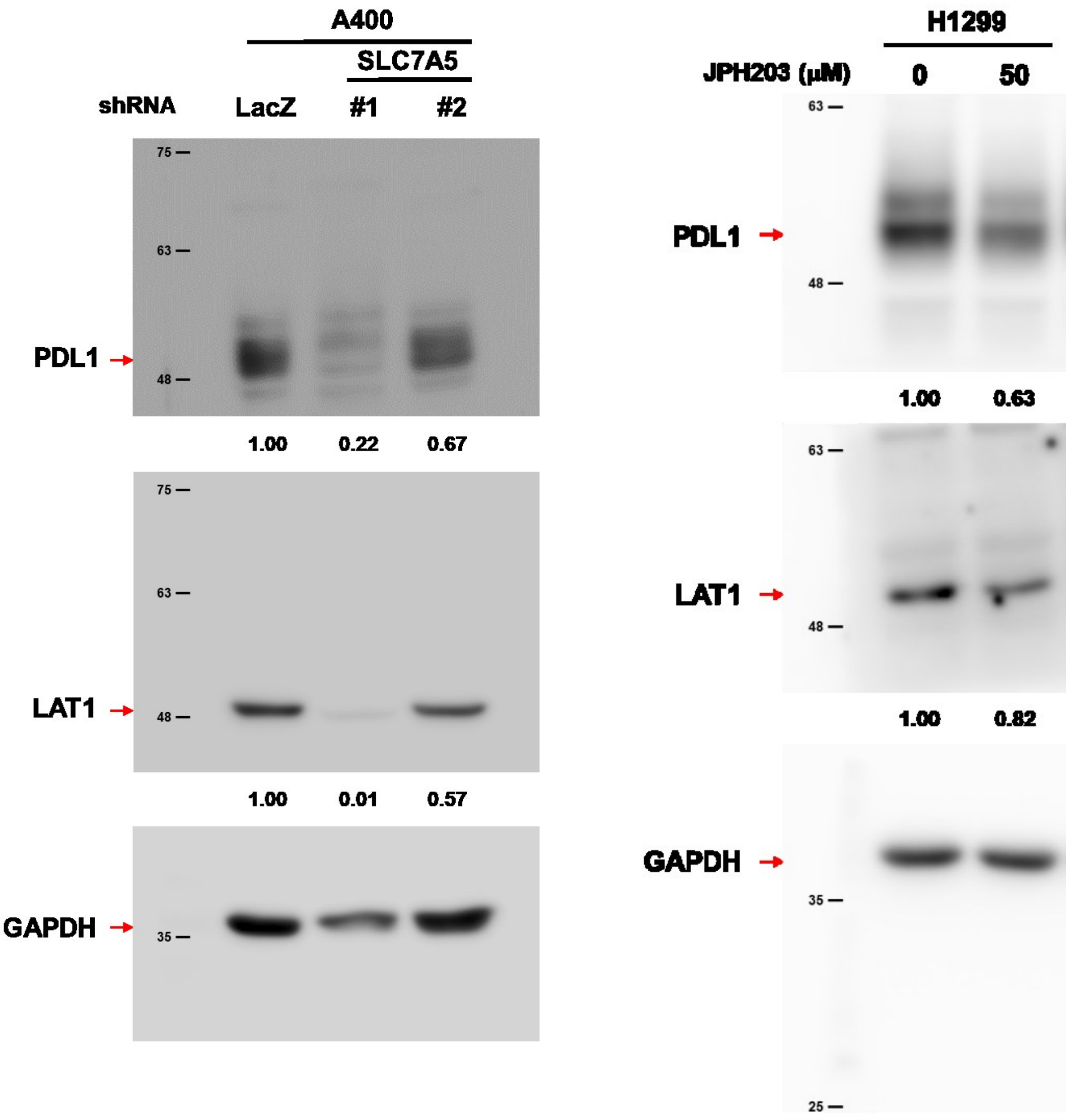

Figure S11. The original blot images of Figure 4B.

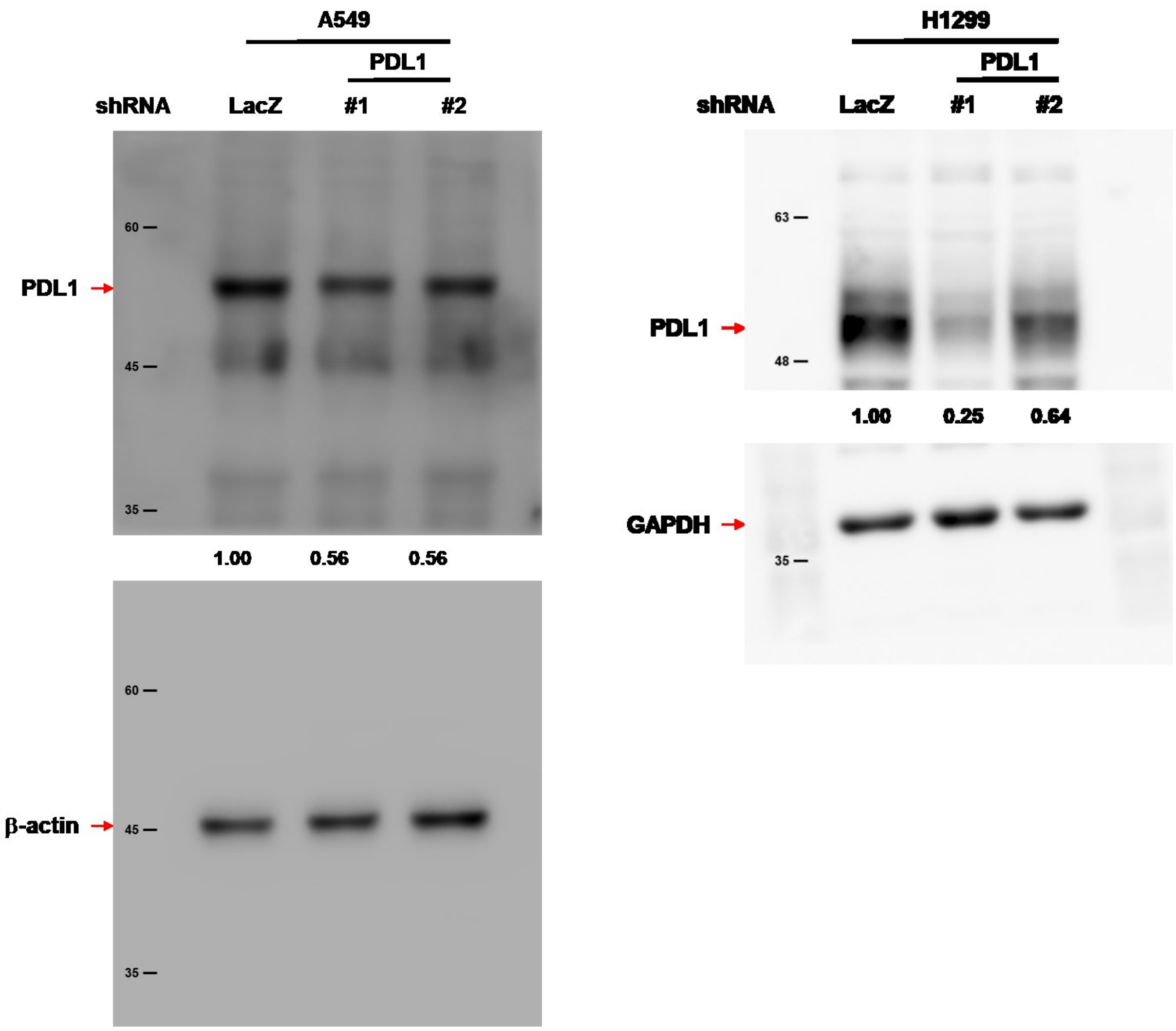

Figure S12. The original blot images of Figure 5B.

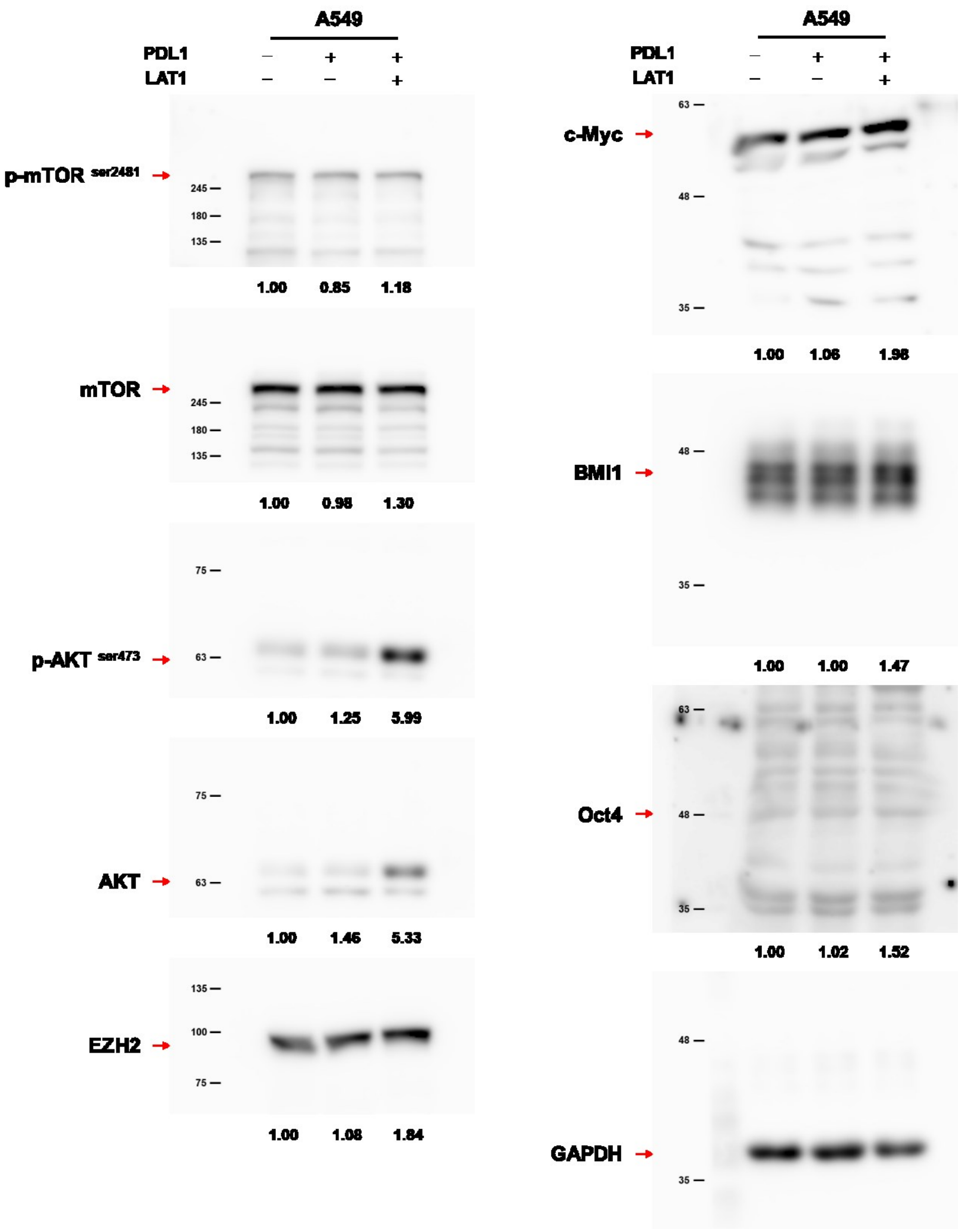

Figure S13. The original blot images of Figure 6C.

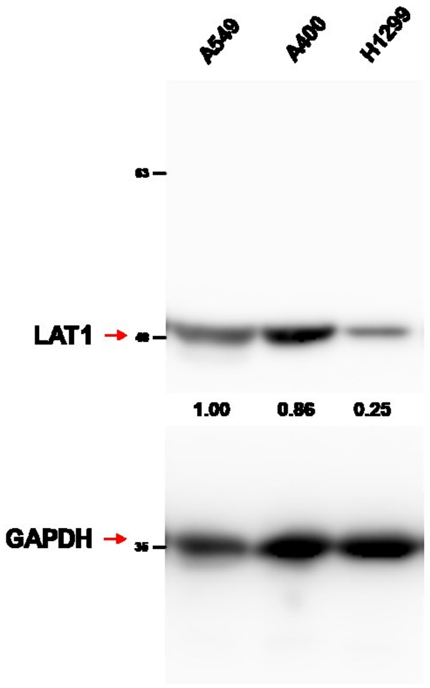

Figure S14. The original blot images of Figure S1.
